# Structural and free energy landscape of novel mutations in ribosomal protein S1 (rpsA) associated with pyrazinamide resistance

**DOI:** 10.1038/s41598-019-44013-9

**Published:** 2019-05-16

**Authors:** Muhammad Tahir Khan, Abbas Khan, Ashfaq Ur Rehman, Yanjie Wang, Khalid Akhtar, Shaukat Iqbal Malik, Dong-Qing Wei

**Affiliations:** 1 Department of Bioinformatics and Biosciences, Capital University of Science and Technology, Islamabad, Pakistan; 2 College of Life Sciences and Biotechnology, The State Key Laboratory of Microbial Metabolism, Shanghai Jiao Tong University, Shanghai, China; 3 National University of Science and Technology, Islamabad, Pakistan

**Keywords:** Computational models, Bacterial infection, Computational models, Bacterial infection

## Abstract

Resistance to key first-line drugs is a major hurdle to achieve the global end tuberculosis (TB) targets. A prodrug, pyrazinamide (PZA) is the only drug, effective in latent TB, recommended in drug resistance and susceptible *Mycobacterium tuberculosis* (MTB) isolates. The prodrug conversion into active form, pyrazinoic acid (POA), required the activity of *pncA* gene encoded pyrazinamidase (PZase). Although *pncA* mutations have been commonly associated with PZA resistance but a small number of resistance cases have been associated with mutationss in RpsA protein. Here in this study a total of 69 PZA resistance isolates have been sequenced for *pncA* mutations. However, samples that were found PZA resistant but *pncA* wild type (*pncA*^WT^), have been sequenced for *rpsA* and *panD* genes mutation. We repeated a drug susceptibility testing according to the WHO guidelines on 18 *pncA*^WT^ MTB isolates. The *rpsA* and *panD* genes were sequenced. Out of total 69 PZA resistant isolates, 51 harbored 36 mutations in *pncA* gene (GeneBank Accession No. MH46111) while, fifteen different mutations including seven novel, were detected in the fourth S1 domain of RpsA known as C-terminal (MtRpsA^CTD^) end. We did not detect any mutations in *panD* gene. Among the *rpsA* mutations, we investigated the molecular mechanism of resistance behind mutations, D342N, D343N, A344P, and I351F, present in the MtRpsA^CTD^ through molecular dynamic simulations (MD). WT showed a good drug binding affinity as compared to mutants (MTs), D342N, D343N, A344P, and I351F. Binding pocket volume, stability, and fluctuations have been altered whereas the total energy, protein folding, and geometric shape analysis further explored a significant variation between WT and MTs. In conclusion, mutations in MtRpsA^CTD^ might be involved to alter the RpsA activity, resulting in drug resistance. Such molecular mechanism behind resistance may provide a better insight into the resistance mechanism to achieve the global TB control targets.

## Introduction

According to the World Health Organization report 2018, 23% of the world’s population (1.7 billion), are infected with latent TB^1^. Among the anti-tuberculosis agents, pyrazinamide (PZA) is the only drug that kills MTB in the latent state which has successfully reduced the time span of TB therapy from 9 to 6 months^2–4^. PZA is a prodrug that depends on MTB encoded, pyrazinamidase (PZase) activity for conversion into pyrazinoic acid (POA), an active form of PZA that targets the trans-translation^5,6^ by interfering with ribosomal protein S1 (RpsA), where POA has been disrupting the transfer messenger RNA complex formation with RpsA (RpsA-tmRNA)^3,7–9^.

In MTB, the RpsA protein has four S1 domains (36–105, 123–188, 209–277, and 294–363)^10^. Residues from 292–363, forming fourth S1 domain which is also known as C-terminus domain of RpsA (MtRpsA^CTD^), is highly conserved among MTB strains and fully capable with POA binding. Amino acids at positions, F307, F310, H322, D352, and Arg357 are present in RNA binding sites of fourth S1 domain^11^. PZA resistance emerges due to mutations at MtRpsA^CTD^ of mycobacterial species, causing conformational changes in the POA binding site^3^. Residues, Lys303, Phe307, Phe310, and Arg357, also known as tmRNA binding site, have been identified, involving in the interactions with two POA molecules^3,12^. In *Mycobacterium smegmatis*, Alanine deletion at the C-terminal (RpsA^ΔA438^) results in the lack of binding with RpsA. MtRpsA^CTD^ is the POA binding site where tmRNA bind each other to form a complex for initiation of translation^12^. Protein structure may have drastic effects due to amino acid substitution, altering the protein structure and function, especially in the active site or binding pockets^13–15^. Mutation may also produces effects at a long-range position^16^. Exploring the mechanism of such changes behind a mutation, has been investigated for better understanding of a particular phenomenon. However, these are time-consuming and very expensive investigations when addressed by experimental procedure alone.

Molecular dynamic (MD) simulations is a method of choice that have been applied widely in exploring the mechanisms of conformational changes in protein, especially in drug resistance mechanisms caused by mutations. MD simulation studies of ligand-protein interactions are widely applied approach to explain the mechanisms of drug resistance due to mutations especially in target protein, which is one of the major causes behind resistance. *In vivo* experimental research, the crystal structure is analyzed for drug resistance while in comparison with experimental approach, MD simulation has a particular advantage to explain the mechanisms of drug resistance at molecular level^14,17^. Further, the structural dynamics of protein complexes and other residues level information can be accessed through MD which have been considered difficult by experimental procedures^18–21^.

In our recent studies, we identified different mutations in *pncA* gene^22^ (GeneBank Accession No. MH46111) and RpsA^23^, whose molecular mechanism of resistance have been investigated through MD simulation^24–26^. Here, we analyzed the effect of our novel mutations, D342N, D343N, A344P, and I351F, on RpsA activity which have been detected in the conserved region in our previous study^23^ among PZA-resistance isolates. We have investigated the possible changes in the RpsA dynamics, that results due to mutations in MtRpsA^CTD^ which may provide useful information behind the drug resistance.

## Results

The repeating DST results demonstrated that the isolates are resistant to PZA. Further, the resistant samples were also analyzed manually where growth was occurred against the critical concentration of PZA. In the previous study, out of total 69 PZA resistant isolates, 51 harbored 36 mutations in *pncA* gene (GeneBank Accession No. MH46111). The remaining 18 isolates were *pncA*^WT^ PZA resistance isolates. Out of 18 PZA resistants but *pncA*^WT^ isolates, 11 (61%) samples have fifteen non-synonymous mutations, while seven isolates were RpsA^WT^ (Table 1). We did not detect any mutation in panD genes. Mutations, S324F, E325K, G341R, D342N, D343N, A344P, and I351F, were present in the conserved region (292–363) of the *rpsA* gene (Table 1). Mechanism of PZA resistance behind mutations S324F, E325K, and G341R in rpsA have been already investigated in our previous study. Here, we analyzed the mechanism of resistance behind mutation, Asp342Asn, Asp343Asn, Ala344Pro, and Ile351Phe, which have been involved in the conformational changes that might be associated with RpsA activity.

**Table 1:** Variants detected in *rpsA* gene^23^.

SNO	Nucleotide Position	Codon No.	Codon Change	Amino Acid Change	Frequency (No. of strains)
1	76delA	26	ATA	Ile26FRAME	1
2	220G>A	74	GTC>ATC	Val74Ile	1
3	278A>G	93	AAG>AGG	Lys93Arg	1
4	618G>A	206	TTG>TTA	Leu206Leu	2
5	636A>C	212	CGA>CGC	Arg212Arg	2
6	830A>G	277	AAG>AGG	Lys277Arg	1
7	971C>T	324	TCC>TTC	Ser324Phe	1
8	973G>A	325	GAG>AAG	Glu325Lys	3
9	1021G>C	341	GGC>CGC	Gly341Arg	1
10	1024G>A	342	GAC>AAC	Asp342Asn	4
11	1027G>A	343	GAC>AAC	Asp343Asn	6
12	1030G>C	344	GCG>CCG	Ala344Pro	6
13	1051A>T	351	ATC>TTC	Ile351Phe	3
14	1108A>C	370	ACC>CCC	Thr370Pro	1
15	1207T>G	403	TGG>GGG	Trp403Gly	1

### Binding pocket and shape complementarity

**Figure 1:**
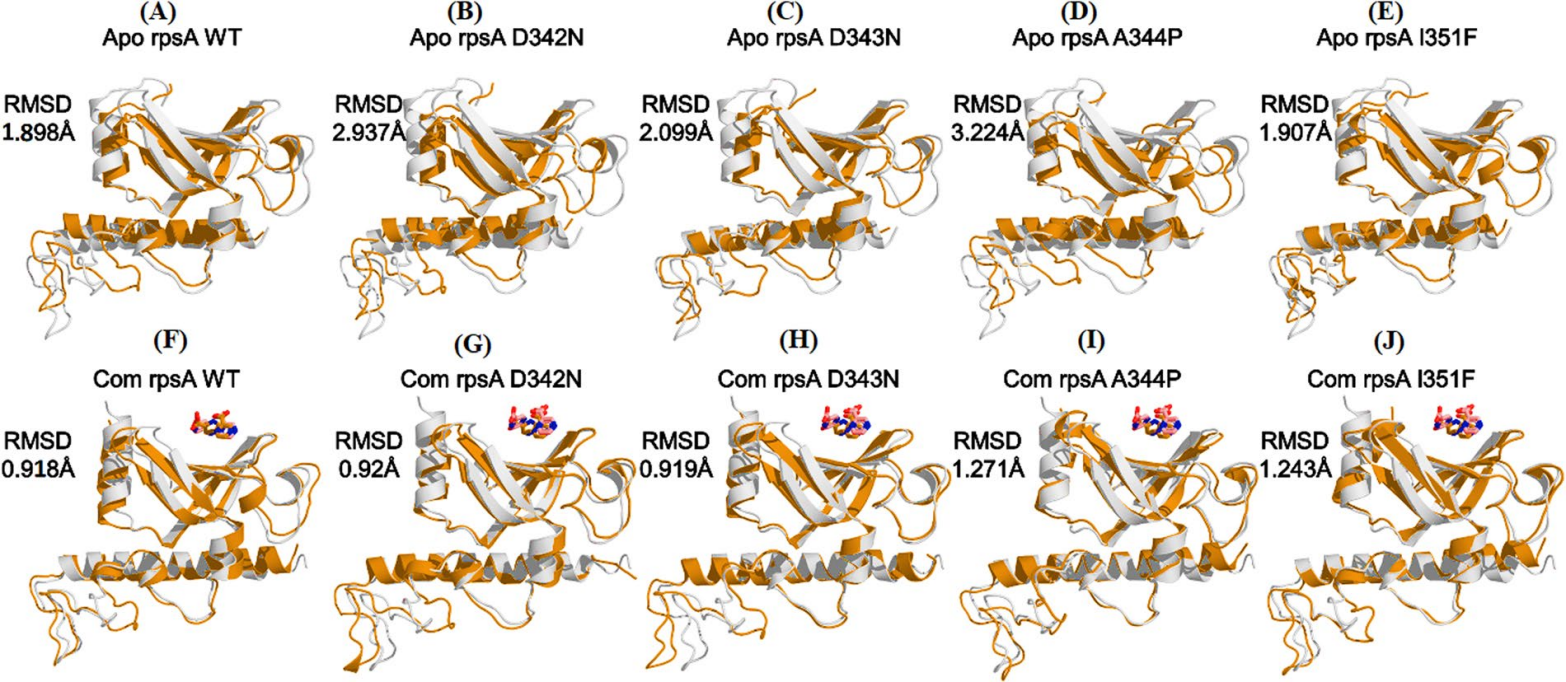
Superimposition of WT-RpsA (**A**) and mutant (**B**–**J**) in apo state and complex (Com) state. RpsA and POA binding residues are 303, 307, 310, and 357. Superimposition of mutant D342N, D343N, A344P, and I351F and WT-RpsA before and after simulation. The difference in RMSDs have been shown with each superimposed structure.

Any diviation from pocket volume may effect the interaction with drug. Compared with WT (4638.493 Å), MTs Asp342Asn (4622.668 Å), Asp343Asn (4623.892 Å), Ala344Pro (4618.064 Å), and Ile351Phe (4623.253 Å), have been altered the binding pocket volume. Shape complementarity score of WT was also found higher (3862) than the MT (Table 2). Further, the RMSD of superimposed MT and WT has a significance difference showing the effect of mutation effect on protein structure (Fig. 1).

**Table 2:** Comparison of PatchDock score, pocket volume, and total binding energy of wild and mutant RpsA.

Cre-Conc * of PZA	DST result	RpsA Mutations	PatchDock score	Pocket Volume (Å)	ΔGbind (Total Binding Energy)
100 µg/ml	Sensitive	Wild type	3862	4638.493	−6.88 kcal/mol
100 µg/ml	Resistant	Asp342Asn	3734	4622.668	−3.408 kcal/mol
100 µg/ml	Resistant	Asp343Asn	3734	4623.892	−3.605 kcal/mol
100 µg/ml	Resistant	Ala344Pro	3738	4618.064	−4.620 kcal/mol
100 µg/ml	Resistant	Ile351Phe	3698	4623.253	−3.937 kcal/mol

Cre-Conc. *, critical concentration.

### Proteins-ligand interactions

**Figure 2:**
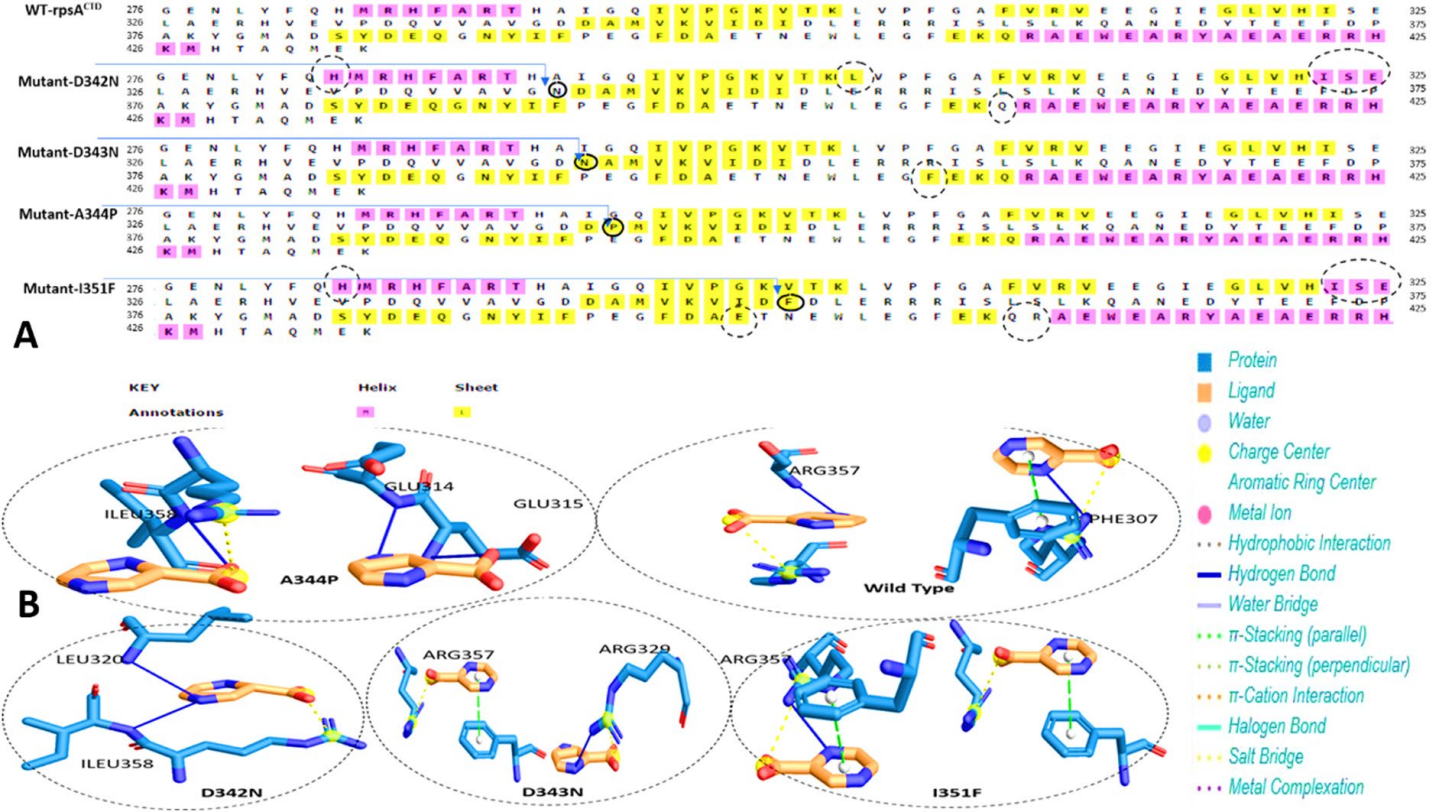
Wild type and mutant’s interactions with POA. (**A**) MTB RpsA WT and MT secondary structure. Mutation site has been pointed, where the conversion of loop into helix and sheet into coil, encircled in D342N, D343N, A344P, and I351F. (**B**) WT formed two H-bonds with two POA molecules. MT had fewer hydrogen as compared to WT.

For a strong binding affinity, hydrogen and hydrophobic are essential interactions between ligand and receptor. Overall residues, R357, F309, G319, L320, A287, I358, D352, D350, S359, L353, F307, F310, E318, and K303 have been involved in the interactions. WT formed more interactions than MTs (Fig. 2).

### Protein and drug trajectory

RMSD is a measure of structure stability during simulation period. During the period of simulation, substantial higher deviation specify that a molecule may be unstable. In apo-state, WT RpsA attained RMSD value between 1.6 Å and 4.0 Å at 70 ns and 15–20 ns repectively. However, the RMSD is seemed to be consistent at 3 Å from 25 to 100 ns. In comparison with WT, MTs, D342N and D343N attained RMSD between 1.5 Å, 3.5 Å and 1.3 Å and 4.3 Å respectively. The RMSD of D343N is seemed to be inconsistent after 75 ns. MT A344P and I351F attained RMSDs between 1.5 Å, 3.5 Å.and 1.0 Å, 4 Å respectively. However, the RMSDs of mutant I351F and A344P are seemed to be unstable as it still in rise at 100 ns.

**Figure 3:**
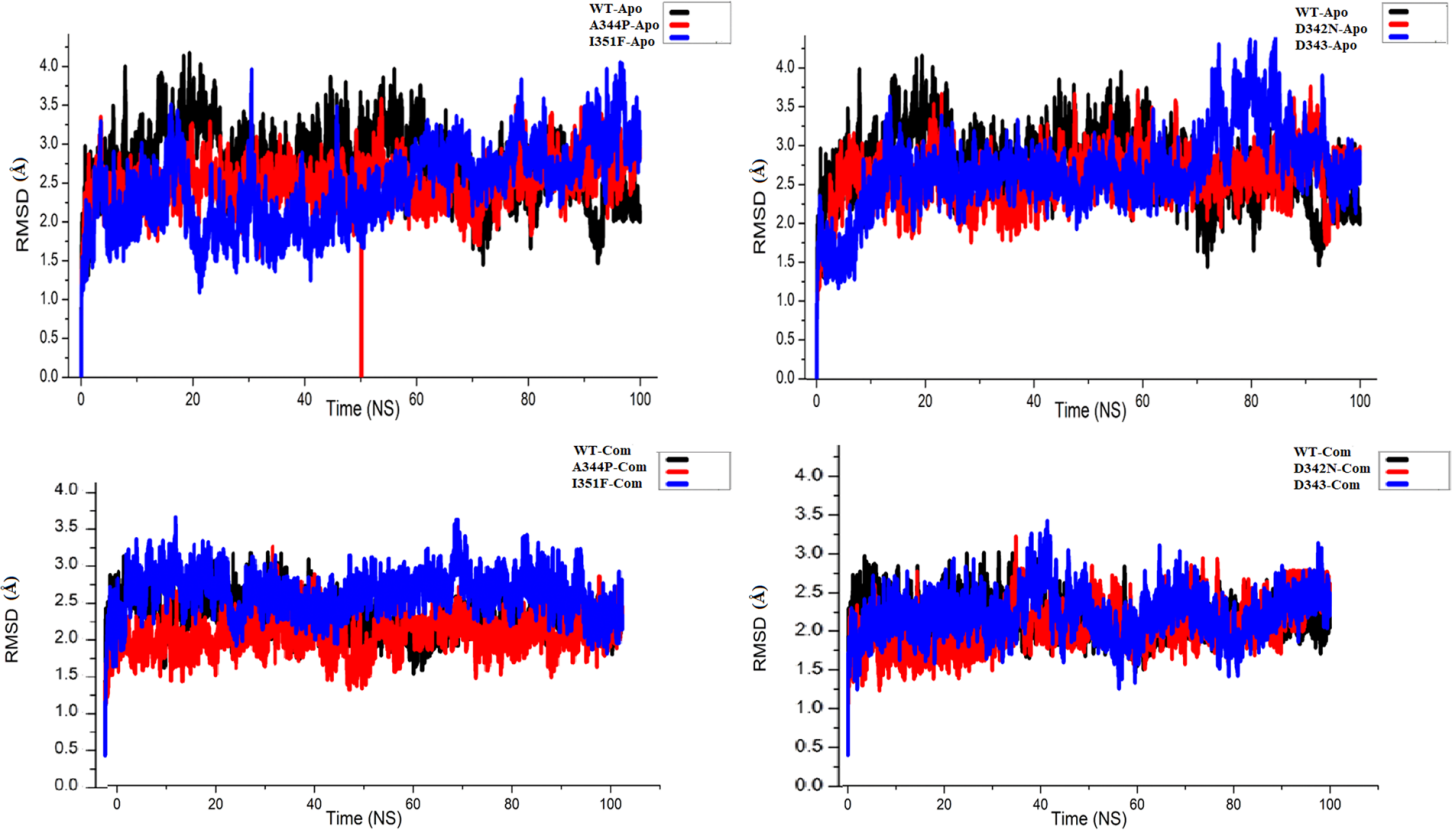
RMSD of wild type and mutants RpsA. Apo state (**A**–**D**) Complex with PZA (**E**–**H**). RpsA^(WT)^; ribosomal protein S1 (RpsA) WT (black) retained a constant RMSD from 25 ns to 100 ns. RMSD of D342N seems to be inconsistent as it rises at 100 ns. In apo state, D343N exhibited a continuous rise in RMSD from 0 ns to 78 ns. However, a little fall have been shown at 95 ns–100 ns. While in complex state, the RMSD is little stable but seems, still rising at 83 ns–100 ns. RMSD of MT A344P is rising at 80 ns–100 ns in apo state, however, it seems stable in complex state. MT I351F attained a little higher RMSD in both, apo and complex state. However, it seems highly unstable in apo state, rising continuously 20 ns–100 ns.

In complex with POA, the RMSD of WT RpsA is seemed to be consistent (1.5 Å–3.2 Å) throughout simulations. The MTs, D342N (1.1 Å–3.5 Å), D343N (1.0 Å–3.5 Å), A344P (1.2 Å–3.4 Å), and I351F (1.2 Å–3.3 Å) exhibited variations in RMSDs during the simulation period (Fig. 3). The MT, D343N and I351F are more deviated at 1000 ns, while, MTs D342N and D343N have been in rise at 100 s.

**Figure 4:**
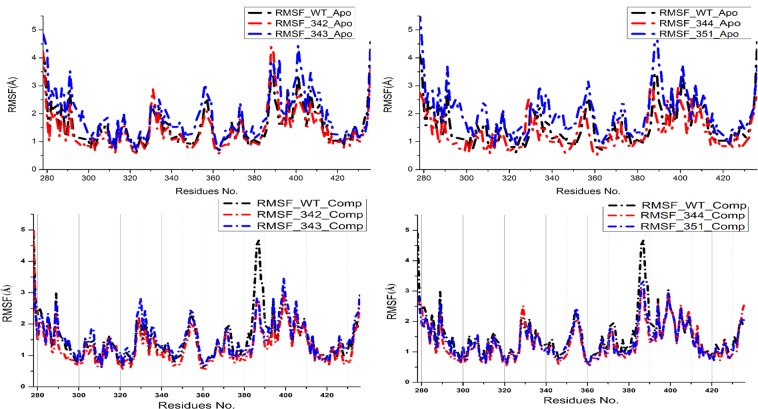
RMSF of wild type and mutant MtRpsA^CTD^. In apo state, residues at locations, 390–410 are more flexible in MT. Residues in D342N exhibited a higher flexibility at lcation 330 and 390. MT, D343N attaind a higher flexibility at position 293, 330, 355, 395–400 in both states. In A344P, the flexibility is seeming almost similar to WT throughout the simulation period. Residues flexibility in MT I351F is higher that WT.

In complex with a drug, RpsA WT exhibited lower flexibility as compared to MTs (Fig. 4). D342N, D343N, and A344P exhibited a higher flexibility at residues lcation 330, 390 and 293, 330, 355, 395–400 in both states. Residues flexibility in MT I351F is higher that WT in apo state.

### Folding dynamics (Radius of gyration (Rg))

**Figure 5:**
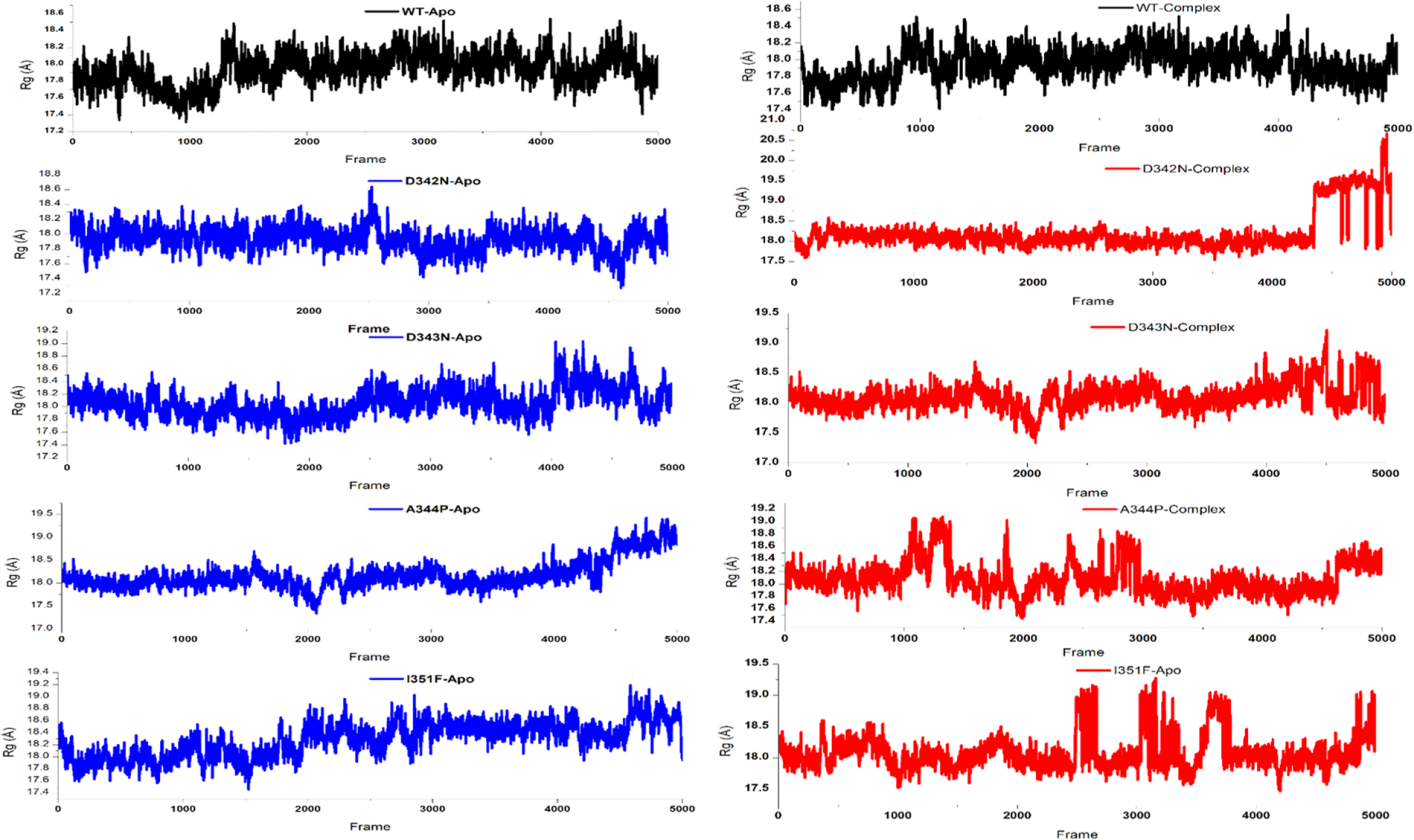
Radius of gyration and B-Factor of wild type and mutant RpsA. A constant Rg value is a measure of correct folding. MT RpsA, D342N, D343N, A344P, and I351F remained unstable throughout simulation period.

The MT exhibited Rg value between 17 and 19 whereas the WT attained the Rg values between 17.4 and 18.3. In both the states, MTs, D342N, D343N, A344P and I351F demonstrated a degree of variation in folding during the simulation period, showing unstable folding than WT. Variations in Rg with time show unstable folding while a stable Rg value indicate compactness in proteins folding. The Rg which measured the degree of compactness and folding is plotted against time. A stable folded protein maintain a steady value of Rg whereas in case of misfolding, the Rg will show variation over time (Fig. 5).

### Essential dynamics

**Figure 6:**
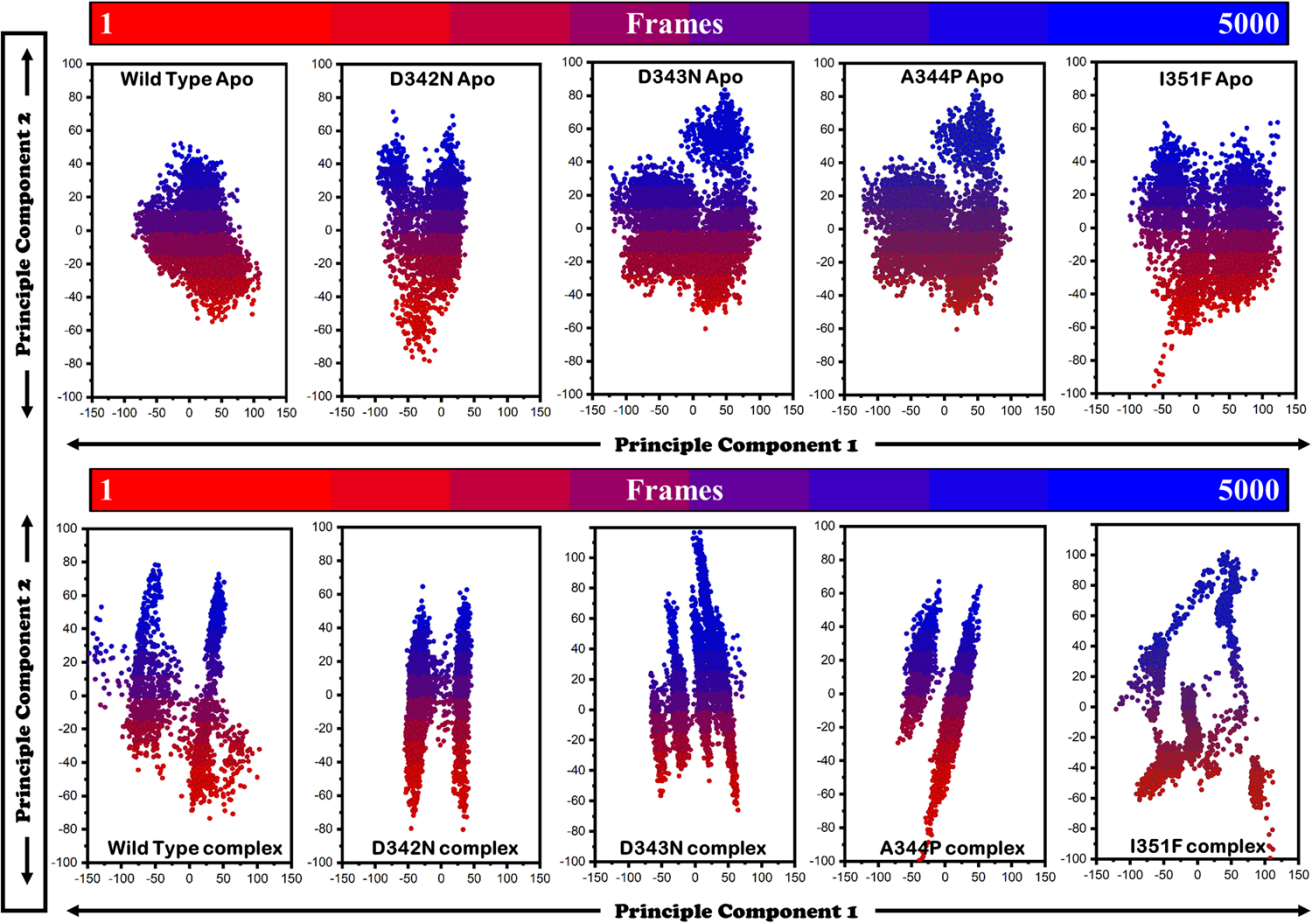
PCA of wild type and mutant RpsA (apo and complex state). MTs RpsA, D342N, D343N, A344P, and I351F scattered over a large area, showing a dispersed type of motion on first two components, PC1 and PC2. These variations in dynamics of WT and MT are more evident in the complex state with the drug. The PC2 motions of MT are significantly more scatered and disperse type, especially I351F.

The mutant’s structures covered more area in both, apo and complex states and is more scattered, showing its variation in dynamics of motions (uncorrelated). PCs of WT and MT have been shown (Fig. 6). MTs, D342N, D343N, A344P, and I351F exhibited a scattered type of motion than WT. In apo state, a cluster type of motion was observed in WT, covering a small area on PC1 and PC2 between −50 and 50, −100 and 100 respectively. MTs RpsA, D342N, D343N, A344P and I351F exhibited a more scattered type of motion on PC1 (between −100 and 50, −125 and 100,–125 and 100, −100 and 125) and PC2 (−80 and 80, −60 and 90, −60 and 90, −100 and 60).

In complex state WT is more dispersed on PC2 (−150 and 100) than mutants D342N, D343N, A344P and I351F (−50 and 50, −75 and 75, −100 and 50, −125 and 125). However, the dynamics of MTs is more scattered (−80 and 70, −70 and 120, −100 and 70, −100 and 100) than WT (−70 and 80).

### Free energy landscape and Gibbs free energy

**Figure 7:**
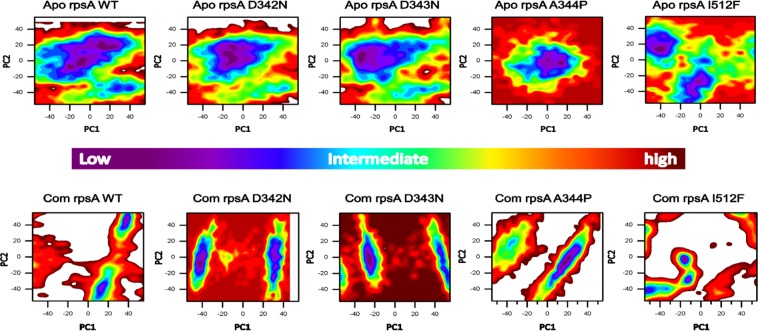
Free energy Landscape (FEL) of the wild type and mutants, D342N, D343N, A344P and I351F. High energy state is represented by red color in the plot. WT has a significant GFE variations in both states (apo and complex) as compared to MT, as indicated by the color of the plot. The color of WT demonstrated more stability compared to MT.

Gibbs free energy (GFE) is a measure of work of a closed system when exchanging heat with the surroundings. The differences in GFE values may have importance in the stability calculation. A protein with native structure has the minimum GFE. WT exhibited a significant difference in GFE than MT, depicted in (Fig. 7). The color (red) in both states of the MT is more unstable compared to the MT.

The ΔG_bind_of WT (−6.88 kcal/mol) is very low in comparison with ΔG_bind_ of D342N (−3.408 kcal/mol), D343N (−3.605 kcal/mol), A344P (−4.620 kcal/mol), and I351F (−3.937 kcal/mol) (Table 2), showing a very low binding affinity and stability. The results of Gibbs free energy and ΔG_bind_ is showing that these mutations might be involved in no or low binding affinity with POA, causing resistance.

### Distance matrix

**Figure 8:**
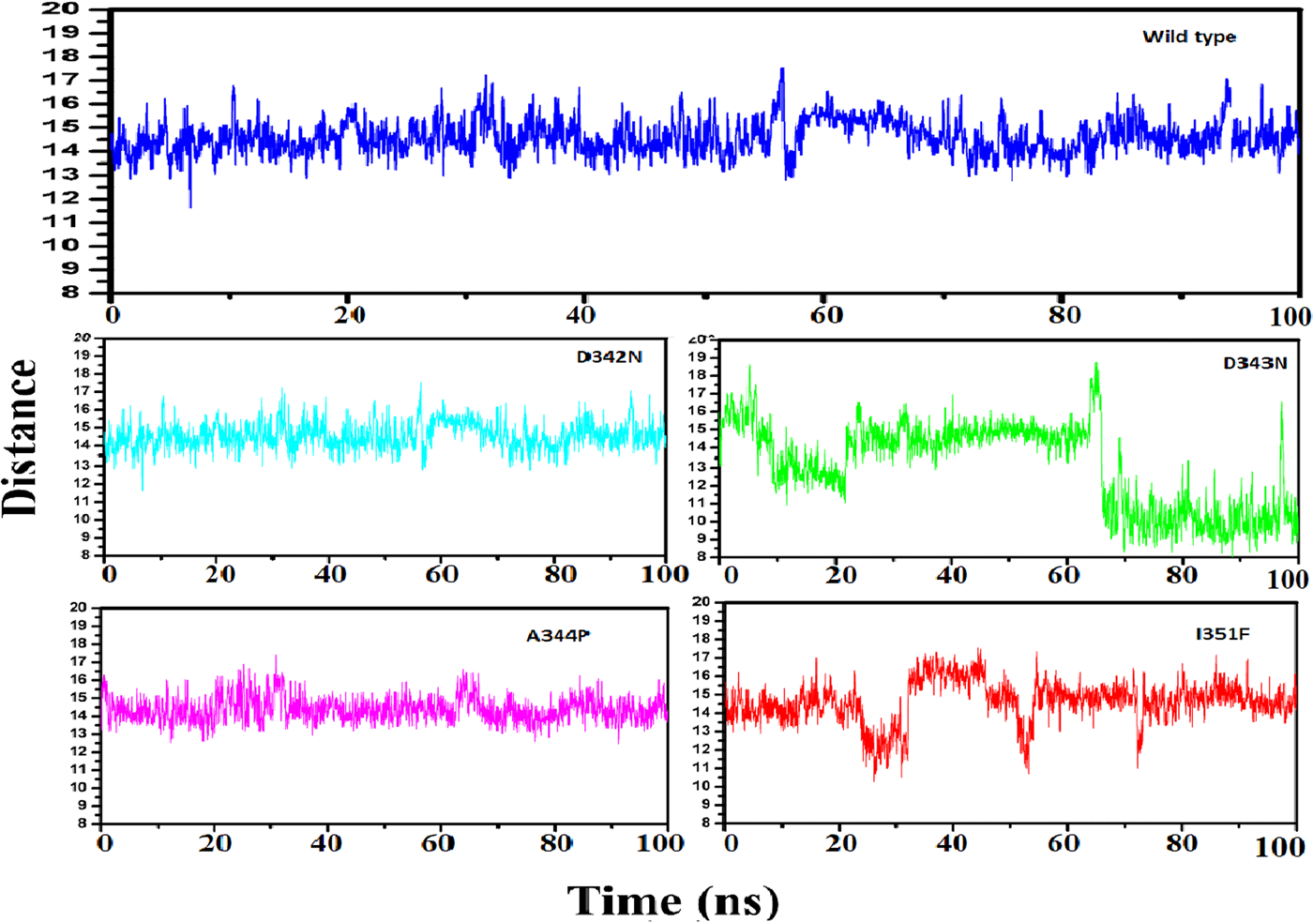
Distance matrix of wild type and mutant RpsA with drug. The average distance of WT and POA is constant. MT, D342N, D343N, A344P, and I351F exhibited variations in distances throughout simulation period. D343N and I351 exhibited much higher distance.

**Figure 9:**
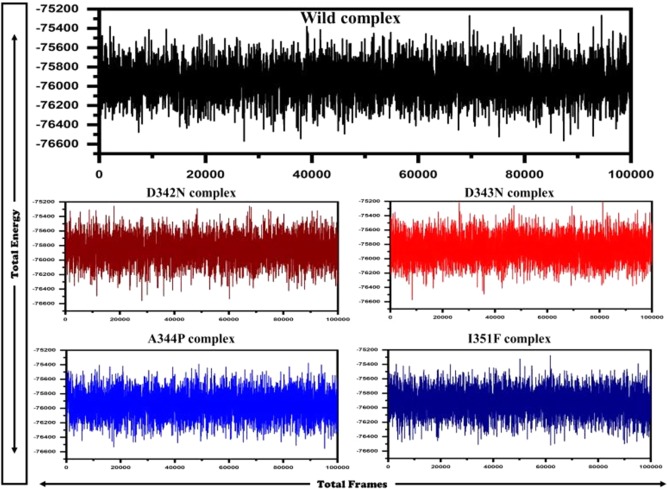
Total estimated energy of wild type and mutant RpsA. Total energy measured for WT was significantly higher than MT D342N, D343N, A344P and I351F.

The average distance of WT RpsA and drug is almost constant during the simulation period as compared to MTs, where a high degree of fluctuation (Fig. 8) representing the variation in distance between drug and protein during the whole simulation period. The MT, D343N and I351F, exhibited a high average distance between target and drug, signifiying the effect of mutations on proteins binding affinity. The total energy of WT RpsA (−76000 kJ/mol) was significantly lower than MTs (74500kj/mol, 73000kj/mol, 71500kj/mol, and 70500kj/mol) throughout simulation as shown in Fig. 9.

## Discussion

Drug resistance is the main obstacle towards the WHO end TB 2030. Investigating the prevalence of PZA resistance in TB high burden countries^23,27^ and insight into drug resistance mechanisms is vital for better supervision of global TB control. PZA is the only drug that kills persister MTB under latent state. According to the previous investigations, the emergence of PZA resistance is associated with mutations in *pncA*, but not in all cases, where resistance may be emerged due to mutations in the target RpsA^23,28–30^. In our previous study^23^, we reported novel mutations in conserved region called MtRpsA^CTD^ (Table 1). However, the molecular mechanism behind resistance was still unknown. Here, we explored the inside mechanism of resistance due to RpsA mutations which have been associated *in-vivo* PZA resistance. MD simulations provide the interaction mechanisms at the molecular level^17,31,32^ along with structural and dynamical information, which is difficult to be determined by experimental procedures. Protein’s structure and function are maintained by conserved region residues where mutations have been reported, leading to conformational changes or a loss of function^33–35^. To explore these mechanistic effects behind the PZA resistance, we analyzed multiple characteristics of protein affected by conserved region mutations in MtRpsA^CTD^.

A higher RMSD and RMSF values of MT, D342N, D343N, A344P, and I351F signifies the effect of point mutations when compared with WT, affecting the activity of RpsA to interact with the POA. Stability and flexibility are essential properties maintaining the activity of biomolecules^36^. Mutations in drug target often affect these properties, making them a vulnerable target to interact with drugs. Increases in residue flexibility have a significant effect on activity. The earlier reports^32,37–39^ have also found the effect of mutations in a specific location, resulting in the loss of activity. Thus RMSD and RMSF may be as a measure of effect behind mutations in targets, resulting in drug resistance or disease where stability is a fundamental property, affecting the function, activity, and regulation of biomolecules. Changes in stability and flexibility of targets proteins may be the loss of thermodynamic stability and protein folding^40^. Destabilization in folding and thermodynamic stability may affect the total energy of biomolecules (Fig. 9).

Further, proteins folding stability may also be measured by Radius of gyration (Rg) which is a degree of compactness, commonly measured in MD simulation by a ratio of the accessible surface area to the surface area of the ideal sphere of the same volume, plotted against time. A stable Rg value signifies the proteins correct folding (Fig. 5), while any deviation is regarded as folding instability^41–43^. In comparison with MTs, a more stable Rg attanied by WT is an indication of correct folding during simulations (Fig. 5).

Oftenly, mutations may occurr far from the active site, causing some drastic changes in binding pocket volume. These distant sites may have a good communication with enzyme function in signal transmissions from one functional site to far site through a series of pathways during biological function^44,45^. Any change to the binding pocket may lead to the loss of interaction with inhibitors. Resistance to the drug may be developed due to change in the binding pocket^38,46,47^. The binding affinity of RpsA to POA may also be lost due to alteration in pocket size. In the binding pocket, the primary interactions accounted with ligand are, hydrogen bonds, van der Waals and electrostatic forces^36^. The three-dimensional structure of a protein has hydrogen and hydrophobic interactions playing a vital role in interactions. Weak interactions have fewer hydrogen and hydrophobic interactions. WT protein has more and MT, D342N, D343N, A344P, and I351F, has significantly fewer interactions indicating the strength of protein folding andbinding stability. Hydrogen bonds support the core, which is comprised of α-helices and β-sheets^48–50^. In spite of mutations, a low level of resistance may be developed due to efflux or influx^51^. Fore more better understanding of drug resistance, the role of the efflux needs to be specified.

We analyzed the consequences of our novel mutations D342N, D343N, A344P, and I351F on RpsA dynamics by comparing them with WT in PZA-resistant *pncA*^*WT*^ MTB strains. These mutations have been involved, changing the RpsA activity by alltering the total energy, flexibility, folding and stability, thereby affecting the interactions with POA. In comparison with WT, interaction of POA and RpsA seemed to be altered due to variations in the binding pocket of MTs D342N, D343N, A344P, and I351F. The overall investigations supports the hypothesis that mutaions in the conserved region of the *rspA* gene might be involved in PZA-resistance. To the best of our knowledge, we presented a first comprehensive investigation of such kind where multiple characteristics have been investigated for better insight into mutations affecting RpsA activity and caused PZA resistance.

## Materials and Methods

### Samples collection

Provincial Tuberculosis Reference Laboratory (PTRL) is the Central laboratory of Khyber Pakhtunkhwa (KPK) province of Pakistan. We sequenced 69 PZA resistance isolates for pncA gene mutations, out of which 51 has 36 different mutations in *pncA* gene^22^ while 18 isolates were detected as *pncA*^WT^. We collected all the 18 isolates, previously detected as resistant to PZA (PZA-R) but *pncA*^WT^. To screen mutation in rpsA and panD genes, all these samples were grown on 7H9 media in the mycobacterium growth indicator tube (MGIT) 960 system for confirmation as MTB^52^. Approximately 100 µl of the sample of the positive tube was added to TBc ID device that indicates a positive MTB by the emergence of pink to red at the test and control location, confirming the antigen, MPT64 in the sample^53,54^. All the positive MTB tubes were subjected for repetition of drug susceptibility testing (DST).

### PZA DST

MTB Drug susceptibility testing (DST) is now routinely performed through automated BACTEC MGIT 960 system^55^. The samples were repeated for PZA DST along with positive (ATCC 25618/H37Rv) and negative controls (*Mycobacterium bovis*). A sample was marked as PZA-resistance when growth occurred at 100 μg/ml of PZA critical concentration^55^.

### Interpretation ofsci drug susceptibility testing

The test completion is interpreted when the growth unit (GU) value of control (GC) attained as 400 or more. An inventory report is printed as “S” for sensitive isolate and “R” for resistance. The growth unit (GU) value was less than 100 for sensitive isolates and greater than 100 for resistants^55–57^.

### *rpsA* and *panD* gene sequencing

DNA was isolated from the PZA resistance samples using the sonication method^58,59^. Forward and reverse primers, F-5′CGGAGCAACCCAACAATA-3′ and R-5′ GTGGACAGCAACGACTTC-3′)^60^ and F-5′TCAACGGTTCCGGTCGGCTGCT-3′, R-5′TATCCGCCACTGCTGCACGACCTT-3′^61^ were used to amplify *rpsA* and panD genes. Each 50-µl reaction consist of molecular grade water (34.8 µl), genomic DNA (4 µl), DNTs (0.1 µl), MgCl2 (3 µl), polymerase chain reaction (PCR) buffer (5 µl), Taq (0.8 µl) (New England Biolabs, UK)), forward and reverse primers (1 µl each). The PCR conditions were adjusted as, 94 °C (5 minutes), as denaturation step of 30 cycles (30 seconds), 30 seconds (56 °C), and for 1 minute (72 °C); 72 °C for 5 minutes as an extension step. The amplified *rpsA* (PCR product) was sent for sequencing (6 Applied Biosystems 3730xl, Macrogen, Korea).

### rpsA sequencing data analysis

The *rpsA* and *panD* sequence data was loaded into Mutation Surveyor V5.0.1^62^ compared with *rpsA* (Rv1630) RefSeq (NC_000962.3) gene.

### Crystal structure retrieval

A crystal structure of MTB RpsA^3^ (PDB ID 4NNI) was retrieved from Brookhaven Raster Display (BRAD) protein data bank (PDB)^63^. Mutant structures of RpsA were generated by inducing mutations at D342N, D343N, A344P, and I351F residues using PYMOL^64^. The POA structure was retrieved from PubChem database (PubChem CID: 1047)^65^ and energy minimized.

### Molecular docking

Structures were loaded into Chimera^66,67^, selenomethionines were changed into methionine and hydrogen atoms were corrected. Shape complementarity measuring score of drug and protein structures was performed using PatchDock server^68^, which is a kind of geometric matching, where drug and protein features are compared for the docking purpose. Molecular complexes have been found with good interactions are oftenly exhibited good shape complementarity. Mutations often cause conformational changes, affecting the interactions with a drug^69,70^. Pocket volumes of WT and mutant D342N, D343N, A344P, and I351F were compared through CastP server^71^.

### Molecular dynamics (MD) simulation

Molecular dynamics (MD) simulations provide plenteous dynamical structural and energetic information about the target protein and drug interactions. The essence of receptor and drug interaction can be analyzed accurately as MD simulation provides useful information in understanding the structure-function relationship, guiding the drug discovery and designing practice^47^. MD simulation was performed on all the structures using AMBER while topological parameters were generated using the antechamber tool (. frcmod). A transferable intermolecular potential with 3 points (TIP3P) water box was used to solvate the proteins and ligand coordinates with a distance of 8.0 Å^72^. Sodium and chloride ions were added to neutralize the systems, as these ions possess high electrostatic potential in the replacement of water molecules. The steepest descent algorithm was used to minimize the energy.

Constant volume and temperature (NVT) ensemble and pressure and temperature (NPT) ensemble was equilibrated and set out at 1-bar pressure for 100 ps. Temperature and pressure were maintained constant using Berendsen thermostat^73^ and Parrinello–Rahman barostat^74^ methods respectively. Linear constraint solver (LINCS) algorithm^75^ was used for bond length rectification and CPPTRAJ was used for the post-dynamics simulation analysis such as RMSD, RMSF, radius of gyration (Rg), and total energy. Further, a free energy landscape calculation methodology, implemented in Gromacs was used to calculate the energy changes and metastable states during the course of simulation.

### Essential dynamics

To obtained internal motion of the system, Principal Component Analysis (PCA) of trajectory was performed on the mass-weighted cartesian coordinates, where rotation and its translation were removed. Low modes are recognize by long dynamics in proteins^76,77^. Further, PCA reduces the motion in a trajectory^78–80^ where A set of variables z1, z2…, zp, transformed, called principal components (PCs) are generated. Free Energy Landscape (FEL)^81,82^ is applied to calculate the energies of macromolecule conformations sets. The first two PCs (PC1 vs. PC2) of PCA computed, give the trajectories on the two principal components of motion^83^. The amplitude of fluctuations in proteins were captured through PCA and the first two components (PC1 and PC2) were plotted to analyze their fluctuation boundries^84,85^.

The lowest energy stable state is represented by the free energy landscape (FEL). The stable state with minimal energy on a plot is shown by deep valleys while boundaries between the deep valleys represent the intermediate conformations^86^. FEL was plotted, using g_sham module while PC1 and PC2 were used to analyze the FEL using the equation:

$${\rm{\Delta }}G(PC1,PC2)=-\,{K}_{B}TlnP(PC1,PC2)$$

Here, PC1 and PC2 are the reaction coordinates, KB represents the Boltzmann constant and P (PC1, PC2) is the first two principal components probability distribution of the system.

### The Gibbs and total binding free energy

Gibbs free energy (G), is a single value, combining enthalpy and entropy while the change in free energy (ΔG) is the sum of the enthalpy plus the product of entropy and the temperature of the system.

The G is minimized to the chemical equilibrium state of the system and it is a thermodynamic potential at constant pressure and temperature, where a reduction is a vital state for processes. The G (Sugita & Kitao, 1998) was plotted against WT. The binding free energy was measured from 500 snapshots at the end of 10-ns trajectory. The free energy of each component was estimated using the following equation:

$${\rm{\Delta }}{{\bf{G}}}_{{\bf{b}}{\bf{i}}{\bf{n}}{\bf{d}}}={{\bf{G}}}_{{\bf{c}}{\bf{o}}{\bf{m}}{\bf{p}}{\bf{l}}{\bf{e}}{\bf{x}}}-({{\bf{G}}}_{{\bf{r}}{\bf{e}}{\bf{c}}{\bf{e}}{\bf{p}}{\bf{t}}{\bf{o}}{\bf{r}}}+{{\bf{G}}}_{{\bf{l}}{\bf{i}}{\bf{g}}{\bf{a}}{\bf{n}}{\bf{d}}})$$

∆G_bind_ represents the total binding free energy The free energy of each component was estimated as:

$${\bf{G}}={{\bf{G}}}_{{\bf{b}}{\bf{o}}{\bf{n}}{\bf{d}}}+{{\bf{G}}}_{{\bf{e}}{\bf{l}}{\bf{e}}}+{{\bf{G}}}_{{\bf{v}}{\bf{d}}{\bf{W}}}+{{\bf{G}}}_{{\bf{p}}{\bf{o}}{\bf{l}}}+{{\bf{G}}}_{{\bf{n}}{\bf{p}}{\bf{o}}{\bf{l}}}-{\bf{T}}{\bf{S}}$$

G_bond_, represents bonded, G_ele_ is electrostatic, and G_vdW_ is van der Waals interactions while G-_pol_ and G_npol_ represent polar and nonpolar to the total solvated free energies.
